# Immediate risk of non-cancer deaths after a cancer diagnosis

**DOI:** 10.1186/s12885-021-08707-6

**Published:** 2021-08-27

**Authors:** Pengcheng Yang, Yongqiang Zheng, Jiayuan Chen, Haotian Ma, Kaixu Yu, Ying Chen, Yun Yang, Bian Wu

**Affiliations:** 1 Division of Gastroenterology, Union Hospital, Tongji Medical College, Huazhong University of Science and Technology, Wuhan, 430022 China; 2 Cancer Center, Union Hospital, Tongji Medical College, Huazhong University of Science and Technology, Wuhan, 430022 China; 3 State Key Laboratory of Oncology in South China, Sun Yat-sen University Cancer Center, Sun Yat-sen University, Guangzhou, 510060 China

**Keywords:** Cancer diagnosis, Non-cancer deaths, Comorbidity, SEER program, Standardized mortality ratios (SMRs)

## Abstract

**Background:**

Receiving a cancer diagnosis may trigger immediate fatal non-cancer health outcomes in addition to dying of cancer itself. We aim to investigate the full pattern of non-cancer deaths in patients within a year of a cancer diagnosis.

**Methods:**

Patients diagnosed with cancer between 1990 and 2016 were identified from the SEER program. Standardized mortality ratios (SMRs) were calculated to characterize the relative risks of non-cancer deaths compared with the general population.

**Results:**

Among 7,366,229 patients, 241,575 non-cancer deaths (15.9%) were recorded in the first year following a cancer diagnosis. The relative risk of non-cancer deaths was 2.34-fold (95% confidence interval (CI): 2.33–2.35) that of the general population. The majority of non-cancer deaths were caused by cardiovascular diseases (21.8%), followed by infectious diseases (7.2%). Significant elevations in mortality risks were observed for nearly all non-cancer causes, particularly in infectious diseases (SMR: 5.08; 95% CI: 5.03–5.13). Patients with liver cancer (SMR: 12.29; 95% CI: 12.06–12.53) were at the highest risk of early non-cancer deaths. The risks of non-cancer deaths were highest within the first month after diagnosis, and decreased rapidly thereafter.

**Conclusions:**

Risks of non-cancer deaths vary by the types of causes and anatomic sites of cancer. Our data underscore the importance of close observation and early multidisciplinary care for noncancer conditions in patients who have recently received a cancer diagnosis.

## Introduction

As recent progress in cancer prevention, diagnosis, and treatment has prolonged survival of patients with cancer, risk of non-cancer deaths is now becoming a great threat to the health of cancer survivors [1]. There are several reasons for this. First, as cancer is largely a disease of elder persons, cancer survivorship and treatment occur in the context of comorbidities. Secondly, the disease itself and its treatment have substantial impact on the short-term and long-term health of the cancer patients, including organ damage, functional disabilities, as well as secondary malignancies. Thirdly, psychological disabilities such as anxiety, depression, stress, fear of recurrence may also lead to certain adverse health outcomes, such as suicide and cardiovascular disease (CVD) [2, 3]. Although a number of the studies have focused on survivorship of long-term cancer survivors [1, 3, 4], the short-term survivorship following a cancer diagnosis has not been well studied. It has been shown that the relative risks in suicides and CVD were significantly elevated in this particular short peroid, probably due to the high levels of distress and psychiatric symptoms resulting from the cancer diagnosis [3, 4]. However, risks of deaths from other non-cancer causes remain unclear.

Understanding the patterns of death is key to predicting the health care needs of a growing cancer population and in developing a public health strategy. This study intended to comprehensively assess all the non-cancer causes of death within the first year immediately after a cancer diagnosis. It also aimed to identify the subgroups at particular risk of early death from non-cancer causes.

## Materials and methods

### Data sources and study population

A retrospective cohort study was performed using data from the Surveillance, Epidemiology, and End Results (SEER) program. As a system of population-based cancer registries from the National Cancer Institute, this program routinely collects and reports data on cancer demographics, incidence, follow-up data, anatomic sites, morphology, stage, therapy, and socioeconomic status of cancer patients in the United States [5].

**Fig. 1:**
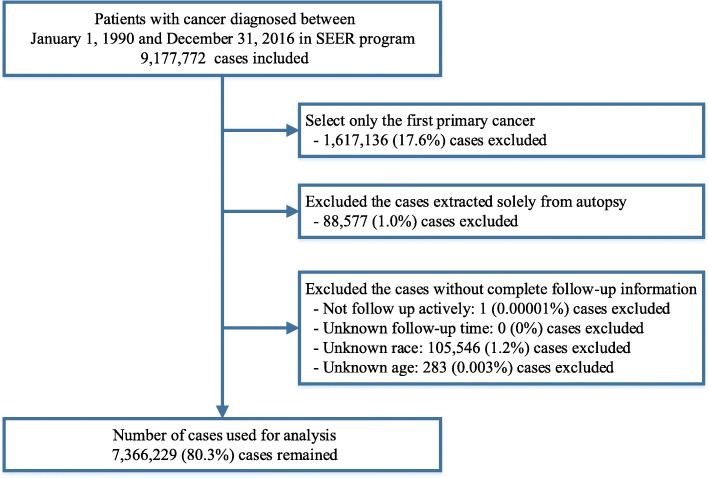
Flow chart of inclusion and exclusion criteria for cases included in this study

All patients diagnosed with cancer between 1990 and 2016 were identified from the SEER 18 database (2019 submission) using the SEER*Stat software version 8.3.6 [5]. Data from patients with only one cancer or those on the first primary cancer were included. Patients were excluded if their diagnosis was obtained exclusively from death certificates or autopsy reports. We further excluded patients without complete follow-up information, including the duration of follow-up, age at diagnosis or race (Fig. 1). To compare with the general population, mortality data of the general US population registered by the National Center for Health Statistics between 1990 and 2016, were also obtained from the SEER database [6].

Since it is a publicly available database, access to the SEER data required a signed research data agreement form. Institutional Review Board approval and informed consent were waived for the data obtained from the SEER database, as the study did not directly involve human subjects, and all data were anonymized.

### Definition of variables

All patients were observed from the time of cancer diagnosis until death within 1 year, exiting the follow-up within 1 year, surviving up to 1 year or the end of the study (December 31, 2016). Non-cancer deaths occurring in the first year after a cancer diagnosis were chosen as the events of interest. For patients who survived more than one year, the first-year follow-up after the cancer diagnosis was taken into account. Among patients included in this study, we evaluated the following variables: age at diagnosis, sex, race, year of diagnosis, marital status, survival in months, cause of death, anatomic site, cancer stage, surgical therapy, chemotherapy and radiotherapy.

Non-cancer deaths (deaths from any medical cause other than cancer) were defined based on SEER cause-of-death classification variables obtained from the death certificates, and were categorized into 26 major groups [7]. The recodes were completed according to the international classification of diseases codes ninth version (ICD-9) for deaths that occurred between 1990 and 1998, or ICD-10 for the deaths that occurred between 1999 and 2016. These groups were further consolidated into 7 broad categories, namely infectious diseases, CVD, respiratory diseases, gastrointestinal and liver diseases, renal diseases, external injuries, and other causes [7, 8]. Although deaths from benign, or unknown behavior neoplasms were also classified as non-cancer deaths by the SEER program, these deaths were not considered as non-cancer deaths during our analyses. The remaining 25 major types of non-cancer cause were included in our analysis.

As the SEER database records the duration of survival in months, and a month was the shortest time interval available for analysis, survival durations shorter than 1 month are recorded as 0 months in the SEER program. Therefore, according to standard epidemiologic conventions, patients with durations of survival coded as zero were converted to half month periods [9].

### Statistical analysis

The mortality rates for non-cancer causes were calculated as the number of non-cancer deaths that occurred within 1 year of diagnosis, divided by person-years of follow-up. For patients who died or exited the study within 1 year from the diagnosis, the actual follow-up duration was used. For patients who survived more than 1 year after diagnosis, the follow-up time was recorded as such. The standardized mortality ratios (SMRs) and corresponding 95% confidence intervals (CIs) of non-cancer deaths were calculated according to previously published methods [9–12]. The SMRs were estimated as the ratios of observed to expected number of non-cancer deaths within the year following a cancer diagnosis. The observed values represented the number of deaths from certain causes in cancer patients, whereas the expected values represented the number of individuals who died of the same causes in the general population, with a similar distribution of age, sex, race, and calendar year. For age and calendar year used in the course of standardization, five-year categories were created and the values at the time of diagnosis were adopted. The race group used in the standardization included White, Black, American Indian/Alaska Native and Asian or Pacific Islander. The 95% CI of the SMRs were obtained using an approximation from the Poisson distribution [9, 13].

All statistical tests were 2-sided, and *P* < .05 were considered statistically significant. Analyses were performed using the SEER*Stat version 8.3.6 (US Department of Health and Human Services) and the R version 3.52 (The R Project for Statistical Computing) statistical software package [5, 14].

## Results

### Baseline characteristics

A total of 7,366,229 patients with cancer were identified for analysis. During follow-up, 1,518,097 deaths were recorded in the first year after diagnosis, of which 241,575 were due to non-cancer causes; this represented 15.9% of total deaths. Compared with the general population, the relative risk of non-cancer deaths in patients with a cancer diagnosis was 2.34 (95% CI: 2.33–2.35) within the first year after diagnosis. Most deaths from non-cancer causes occurring within the first year were observed in patients who were 60 years or older (83.5%), male (58.1%) and white of race (80.7%).

Compared with the general population, non-cancer death risks within the first year after diagnosis were higher among those with an age of 20 to 39 years (SMR: 19.88; 95% CI: 19.43–20.34), of Asian or Pacific Islander ethnicity (SMR: 3.82; 95% CI: 3.62–4.03), unmarried (SMR: 2.92; 95% CI: 2.90–2.93), with distant metastases (SMR: 4.13; 95% CI: 4.10–4.17), not treated with surgery (SMR: 3.57; 95% CI: 3.55–3.59), and not treated with radiotherapy (SMR: 2.60; 95% CI: 2.59–2.61). The baseline characteristics are detailed in Table 1. The mortality from non-cancer causes was higher within the first year (SMR: 2.34; 95% CI: 2.33–2.35) than that after a year, among cancer patients (SMR, 1.81; 95% CI: 1.80–1.81).

**Table 1:** Risk of non-cancer deaths among patients with cancer by demographic and tumor characteristics for the non-cancer deaths occurred within the first year or after a year following diagnosis

Characteristics	Within 1 years since diagnosis					More than 1 year since diagnosis				
	No. of patients with cancer	Person-years of follow up	No. of observed deaths	No. of expected deaths	SMR^a^ (95% CI)	No. of patients with cancer	Person-years of follow up	No. of observed deaths	No. of expected deaths	SMR^a^ (95% CI)
All	7,366,229 (100%)	5,624,828	241,575 (100.0%)	103,105.6	2.34 (2.33–2.35)	5,425,172 (100%)	36,196,870	864,975 (100.0%)	478,513.4	1.81 (1.80–1.81)
Age, y										
0–19	92,844 (1.3%)	78,747	583 (0.2%)	54.7	10.66 (9.83–11.57)	79,811 (1.5%)	669,075	1080 (0.1%)	502.5	2.15 (2.03–2.28)
20–39	442,398 (6.0%)	372,394	7364 (3.0%)	370.4	19.88 (19.43–20.34)	377,627 (7.0%)	3,211,248	11,940 (1.4%)	3160.8	3.78 (3.71–3.85)
40–59	2,234,414 (30.3%)	1,819,643	32,028 (13.3%)	6296.1	5.09 (5.03–5.14)	1,806,936 (33.3%)	13,661,030	97,263 (11.2%)	45,017.2	2.16 (2.15–2.17)
60–79	3,585,461 (48.7%)	2,719,691	111,548 (46.2%)	43,170.3	2.58 (2.57–2.60)	2,606,293 (48.0%)	16,492,660	514,259 (59.5%)	260,950.5	1.97 (1.97–1.98)
80+	1,011,112 (13.7%)	634,352	90,052 (37.3%)	53,214.1	1.69 (1.68–1.70)	554,505 (10.2%)	2,162,857	240,433 (27.8%)	168,882.4	1.42 (1.42–1.43)
Sex										
Female	3,717,559 (50.5%)	2,872,180	101,161 (41.9%)	44,397.1	2.28 (2.26–2.29)	2,794,614 (51.5%)	19,176,433	377,760 (43.7%)	194,247.3	1.94 (1.94–1.95)
Male	3,648,670 (49.5%)	2,752,647	140,414 (58.1%)	58,708.5	2.39 (2.38–2.40)	2,630,558 (48.5%)	17,020,437	487,215 (56.3%)	284,266.0	1.71 (1.71–1.72)
Race										
White	6,068,860 (82.4%)	4,653,123	194,848 (80.7%)	88,602.2	2.20 (2.19–2.21)	4,503,707 (83.0%)	30,498,046	738,242 (85.3%)	410,790.0	1.80 (1.79–1.80)
Black	771,611 (10.5%)	574,467	32,978 (13.7%)	10,606.9	3.11 (3.08–3.14)	542,333 (10.0%)	3,238,518	85,172 (9.8%)	50,241.0	1.70 (1.68–1.71)
AI/AN	40,833 (0.6%)	30,360	1310 (0.5%)	342.7	3.82 (3.62–4.03)	28,634 (0.5%)	179,376	3894 (0.5%)	1535.6	2.54 (2.46–2.62)
API	484,925 (6.6%)	366,878	12,439 (5.1%)	3553.8	3.50 (3.44–3.56)	350,498 (6.5%)	2,280,930	37,667 (4.4%)	15,946.8	2.36 (2.34–2.39)
Year of diagnosis										
1990–1999	1,319,905 (17.9%)	1,019,773	55,329 (22.9%)	22,522.2	2.46 (2.44–2.48)	998,514 (18.4%)	11,120,394	323,784 (37.4%)	149,161.2	2.17 (2.16–2.18)
2000–2009	3,422,077 (46.5%)	2,687,274	112,518 (46.6%)	49,977.6	2.25 (2.24–2.26)	2,675,604 (49.3%)	20,562,263	457,002 (52.8%)	268,827.0	1.70 (1.70–1.70)
2010–2016	2,624,247 (35.6%)	1,917,780	73,728 (30.5%)	30,605.8	2.41 (2.39–2.43)	1,751,054 (32.3%)	4,514,212	84,189 (9.7%)	60,525.2	1.39 (1.38–1.40)
Marital status										
Married	4,022,510 (54.6%)	3,173,567	96,497 (39.9%)	50,042.2	1.93 (1.92–1.94)	3,107,029 (57.3%)	21,986,915	441,489 (51.0%)	266,531.8	1.66 (1.65–1.66)
Unmarried	2,788,366 (37.9%)	2,013,382	129,163 (53.5%)	44,251.0	2.92 (2.90–2.93)	1,881,706 (34.7%)	11,414,836	352,443 (40.7%)	169,938.6	2.07 (2.07–2.08)
Unknown	555,353 (7.5%)	437,878	15,915 (6.6%)	8812.4	1.81 (1.78–1.83)	436,437 (8.0%)	2,795,119	71,043 (8.2%)	42,042.9	1.69 (1.68–1.70)
Stage										
In situ	451,459 (6.1%)	405,558	4425 (1.8%)	5442.4	0.81 (0.79–0.84)	436,799 (8.1%)	3,550,928	57,379 (6.6%)	35,976.8	1.59 (1.58–1.61)
Localized	2,838,911 (38.5%)	2,505,010	57,978 (24.0%)	45,487.3	1.27 (1.26–1.29)	2,656,038 (49.0%)	19,930,354	463,751 (53.6%)	276,381.0	1.68 (1.67–1.68)
Regional	1,189,160 (16.1%)	961,350	40,351 (16.7%)	16,393.8	2.46 (2.44–2.49)	937,482 (17.3%)	5,515,953	127,670 (14.8%)	62,437.5	2.04 (2.03–2.06)
Distant	1,294,149 (17.6%)	732,298	56,706 (23.5%)	13,720.3	4.13 (4.10–4.17)	566,503 (10.4%)	2,055,188	52,578 (6.1%)	25,174.3	2.09 (2.07–2.11)
Unstaged	1,592,550 (21.6%)	1,020,611	82,115 (34.0%)	22,061.8	3.72 (3.70–3.75)	828,350 (15.3%)	5,144,448	163,597 (18.9%)	78,543.8	2.08 (2.07–2.09)
Surgery										
Yes	4,373,773 (59.4%)	3,717,285	79,762 (33.0%)	57,493.4	1.39 (1.38–1.40)	3,783,143 (69.7%)	27,669,481	563,877 (65.2%)	320,575.3	1.76 (1.75–1.76)
No	2,926,045 (39.7%)	1,862,953	159,261 (65.9%)	44,599.3	3.57 (3.55–3.59)	1,602,190 (29.5%)	8,330,829	294,677 (34.1%)	154,778.2	1.90 (1.90–1.91)
Unknown	66,411 (0.9%)	44,589	2552 (1.1%)	1012.8	2.52 (2.42–2.62)	39,839 (0.7%)	196,560	6421 (0.7%)	3159.9	2.03 (1.98–2.08)
Chemotherapy										
Yes	1,947,887 (26.4%)	1,511,435	40,799 (16.9%)	16,992.5	2.40 (2.38–2.42)	1,361,418 (25.1%)	7,262,809	110,938 (12.8%)	53,628.9	2.07 (2.06–2.08)
No/Unknown	5,418,342 (73.6%)	4,113,392	200,776 (83.1%)	86,113.1	2.33 (2.32–2.34)	4,063,754 (74.9%)	28,934,062	754,037 (87.2%)	424,884.5	1.77 (1.77–1.78)
Radiotherapy										
Yes	2,021,621 (27.4%)	1,635,770	32,758 (13.6%)	22,878.1	1.43 (1.42–1.45)	1,568,122 (28.9%)	10,489,447	206,859 (23.9%)	118,384.9	1.75 (1.74–1.75)
No/unknown	5,344,608 (72.6%)	3,989,058	208,817 (86.4%)	80,227.5	2.60 (2.59–2.61)	3,857,050 (71.1%)	25,707,423	658,116 (76.1%)	360,128.5	1.83 (1.82–1.83)

Abbreviations: SMR: standardized mortality ratios; CI, confidence interval; AI/AN: American Indian/Alaska Native; API: Asian or Pacific Islander

^a^ For the categories of age, sex, race and year of diagnosis, the SMR reference population was the specific category in the US subpopulation from 1990 through 2015. For the other variables, the SMR reference population was the entire US population from 1990 through 2015

### Mortality for major types of non-cancer causes

On cause-specific analysis, the first leading non-cancer cause of death was CVD (21.8%), followed by infectious diseases (7.2%), and respiratory diseases (4.4%). Compared with the general population, the SMRs were particularly high for infectious diseases (SMR: 5.08; 95% CI: 5.03–5.13) and gastrointestinal and liver diseases (SMR: 4.62; 95% CI: 4.51–4.73). Although lower, the mortality rates for other causes were higher than that of the general population. Patients with cancer have relatively high SMR from CVD (SMR: 2.02; 95% CI: 2.01–2.04) and suicide (SMR: 2.85; 95% CI: 2.74–2.96) in the first year after diagnosis (Table 2).

**Table 2:** Risk of non-cancer deaths for the 25 major types of non-cancer causes within the first year after diagnosis among cancer patients

Cause	No. of observed deaths	Mortality rate	No. of expected deaths	SMR (95% CI)
**Cardiovascular diseases**	**105,518 (21.8%)**	**1875.9**	**52,130.6**	**2.02 (2.01–2.04)**
Diseases of heart	82,346 (17.0%)	1464.0	39,992.2	2.06 (2.05–2.07)
Cerebrovascular diseases	15,959 (3.3%)	283.7	8718.0	1.83 (1.80–1.86)
Hypertension without heart disease	3039 (0.6%)	54.0	1303.7	2.33 (2.25–2.42)
Aortic aneurysm and dissection	1522 (0.3%)	27.1	865.2	1.76 (1.67–1.85)
Atherosclerosis	1468 (0.3%)	26.1	675.5	2.17 (2.06–2.29)
Other diseases of arteries, arterioles, capillaries	1184 (0.2%)	21.0	573.9	2.06 (1.95–2.18)
**Infectious diseases**	**34,997 (7.2%)**	**622.2**	**6889.5**	**5.08 (5.03–5.13)**
Pneumonia and influenza	9799 (2.0%)	174.2	3849.7	2.55 (2.50–2.60)
Syphilis	3 (0.001%)	0.1	1.1	2.65 (0.85–8.21)
Tuberculosis	157 (0.03%)	2.8	48.9	3.21 (2.75–3.75)
Septicemia	7185 (1.5%)	127.7	1870.2	3.84 (3.75–3.93)
Other infectious and parasitic diseases including HIV	17,853 (3.7%)	317.4	856.8	20.84 (20.53–21.14)
**Respiratory diseases**	**21,229 (4.4%)**	**377.4**	**8188.6**	**2.59 (2.56–2.63)**
Chronic obstructive pulmonary disease and allied cond	21,229 (4.4%)	377.4	8188.6	2.59 (2.56–2.63)
**Gastrointestinal and liver diseases**	**7338 (1.5%)**	**130.5**	**1588.8**	**4.62 (4.51–4.73)**
Stomach and duodenal ulcers	990 (0.2%)	17.6	238.2	4.16 (3.91–4.42)
Chronic liver disease and cirrhosis	6348 (1.3%)	112.9	1349.8	4.70 (4.59–4.82)
**Renal diseases**	**5678 (1.2%)**	**100.9**	**2481.6**	**2.29 (2.23–2.35)**
Nephritis, nephrotic syndrome and nephrosis	5678 (1.2%)	100.9	2481.6	2.29 (2.23–2.35)
**External injuries**	**8875 (1.8%)**	**157.8**	**4755.9**	**1.87 (1.83–1.91)**
Accidents and adverse effects	6009 (1.2%)	106.8	3614.9	1.66 (1.62–1.70)
Suicide and self-inflicted injury	2653 (0.5%)	47.2	931.6	2.85 (2.74–2.96)
Homicide and legal intervention	213 (0.04%)	3.8	208.1	1.02 (0.90–1.17)
**Other non-cancer causes of death**	**57,940 (12.0%)**	**1030.1**	**27,070.3**	**2.14 (2.12–2.16)**
Alzheimers	3146 (0.7%)	55.9	3728.7	0.84 (0.81–0.87)
Diabetes mellitus	7263 (1.5%)	129.1	4093.4	1.77 (1.73–1.82)
Congenital anomalies	446 (0.1%)	7.9	144.8	3.08 (2.81–3.38)
Certain conditions originating in perinatal period	50 (0.01%)	0.9	15.5	3.22 (2.44–4.25)
Complications of pregnancy, childbirth, puerperium^a^	173 (0.04%)	3.1	4.7	36.62 (31.55–42.50)
Symptoms, signs and ill-defined conditions	3921 (0.8%)	69.7	1390.4	2.82 (2.73–2.91)
Other cause of death	42,941 (8.9%)	763.4	17,689.8	2.43 (2.40–2.45)

Abbreviations: SMR, standardized mortality ratio; CI, confidence interval; HIV, human immunodeficiency virus

^a^ For the deaths from complications of pregnancy, childbirth, puerperium, which occurred only in female patients, the SMR reference population was the female general population in the United States from 1990 through 2015. For the other causes, the SMR reference population was the entire US population from 1990 through 2015

### Anatomic sites associated with higher risks of non-cancer deaths

In the site-specific analysis, data are presented only for persons with those forms of cancer in which 20,000 person-years or more of survival time was accrued in the data obtained from the SEER registries. Of the different cancer sites, lung cancer was responsible for the highest number of non-cancer deaths (17.7%), followed by colorectal (12.3%) and prostate cancer (7.3%).

Except for cancers of the breast, prostate, and non-basal skin, mortality risks for most types of cancer within the first year after diagnosis were higher than that of the general population (Table 3). Patients with liver cancer (SMR: 12.29; 95% CI: 12.06–12.53) were at the highest risk of early non-cancer deaths, followed by brain (SMR: 5.51; 95% CI: 5.32–5.71), lung (SMR: 4.47; 95% CI: 4.43–4.52), and esophageal cancer (SMR: 4.18; 95% CI: 4.04–4.32).

**Table 3:** Risk of non-cancer deaths within the first years after diagnosis among patients with cancer by anatomic site

Site^a^	No. of patients with cancer	Person-years of follow-up	No. of observed deaths	No. of expected deaths	SMR (95% CI)
All sites	7,366,229 (100%)	5,624,828	241,575 (100%)	103,105.6	2.34 (2.33–2.35)
Liver	111,247 (1.5%)	55,822	10,224 (4.2%)	831.8	12.29 (12.06–12.53)
Brain	106,651 (1.4%)	70,145	3047 (1.3%)	552.6	5.51 (5.32–5.71)
Lung and bronchus	809,032 (11.0%)	445,176	42,791 (17.7%)	9566.6	4.47 (4.43–4.52)
Esophagus	62,611 (0.8%)	37,184	3291 (1.4%)	788.1	4.18 (4.04–4.32)
Pancreas	167,632 (2.3%)	73,063	6406 (2.7%)	1617.9	3.96 (3.86–4.06)
Lymphoma	319,815 (4.3%)	244,742	16,157 (6.7%)	4123.7	3.92 (3.86–3.98)
Myeloma	88,691 (1.2%)	65,552	5460 (2.3%)	1460.8	3.74 (3.64–3.84)
Stomach	112,398 (1.5%)	67,433	5522 (2.3%)	1599.0	3.45 (3.36–3.55)
Leukemia	186,654 (2.5%)	132,021	8583 (3.6%)	2493.8	3.44 (3.37–3.52)
Cervix uteri^b^	70,499 (1.0%)	57,734	1149 (0.5%)	344.5	3.33 (3.15–3.53)
Cranial nerves other nervous system	123,844 (1.7%)	100,873	4947 (2.0%)	1796.7	2.75 (2.68–2.83)
Anus	31,767 (0.4%)	26,380	882 (0.4%)	323.5	2.73 (2.55–2.91)
Larynx	54,655 (0.7%)	44,687	2191 (0.9%)	807.9	2.71 (2.60–2.83)
Oral cavity and pharynx	160,831 (2.2%)	129,003	5441 (2.3%)	2063.2	2.64 (2.57–2.71)
Ovary^b^	109,496 (1.5%)	81,095	2477 (1.0%)	979.9	2.53 (2.43–2.63)
Kidney and renal pelvis	198,567 (2.7%)	154,145	6206 (2.6%)	2486.7	2.50 (2.43–2.56)
Testis^b^	46,488 (0.6%)	40,099	241 (0.1%)	97.3	2.48 (2.18–2.81)
Colon and rectum	682,370 (9.3%)	530,418	29,597 (12.3%)	13,105.5	2.26 (2.23–2.28)
Soft tissue including heart	45,685 (0.6%)	36,157	1033 (0.4%)	513.2	2.01 (1.89–2.14)
Endocrine system	228,233 (3.1%)	193,951	2110 (0.9%)	1184.5	1.78 (1.71–1.86)
Urinary bladder	261,604 (3.6%)	213,851	11,290 (4.7%)	6537.2	1.73 (1.70–1.76)
Vulva^b^	43,166 (0.6%)	36,857	818 (0.3%)	481.4	1.70 (1.59–1.82)
Corpus uteri^b^	208,523 (2.8%)	173,569	3604 (1.5%)	2167.5	1.66 (1.61–1.72)
Breast	1,221,821 (16.6%)	1,060,827	12,947 (5.4%)	13,537.5	0.96 (0.94–0.97)
Skin, non-basal	457,007 (6.2%)	389,550	5822 (2.4%)	6761.9	0.86 (0.84–0.88)
Prostate^b^	1,044,123 (14.2%)	911,841	17,582 (7.3%)	21,074.9	0.83 (0.82–0.85)

Abbreviations: SMR, standardized mortality ratio; CI, confidence interval;

^a^ To minimize the CIs of site-specific SMR, data are presented only for persons with those forms of cancer in which 20,000 person-years or more of survival time accrued in the data obtained from the SEER registries. The chosen sites represent for over 85% of all cancer patients included in the study

^b^ For patients with cancers of ovary, vulva, corpus uteri and cervix uteri, which occurred only in female patients, the SMR reference population was the female general population in the United States from 1990 through 2015. For patients with cancers of testis and prostate, which occurred only in male patients, the SMR reference population was the male general population in the United States from 1990 through 2015. For the other sites, the SMR reference population was the entire US population from 1990 through 2015

### Trends in SMR of non-cancer deaths based on time following diagnosis

**Fig. 2:**
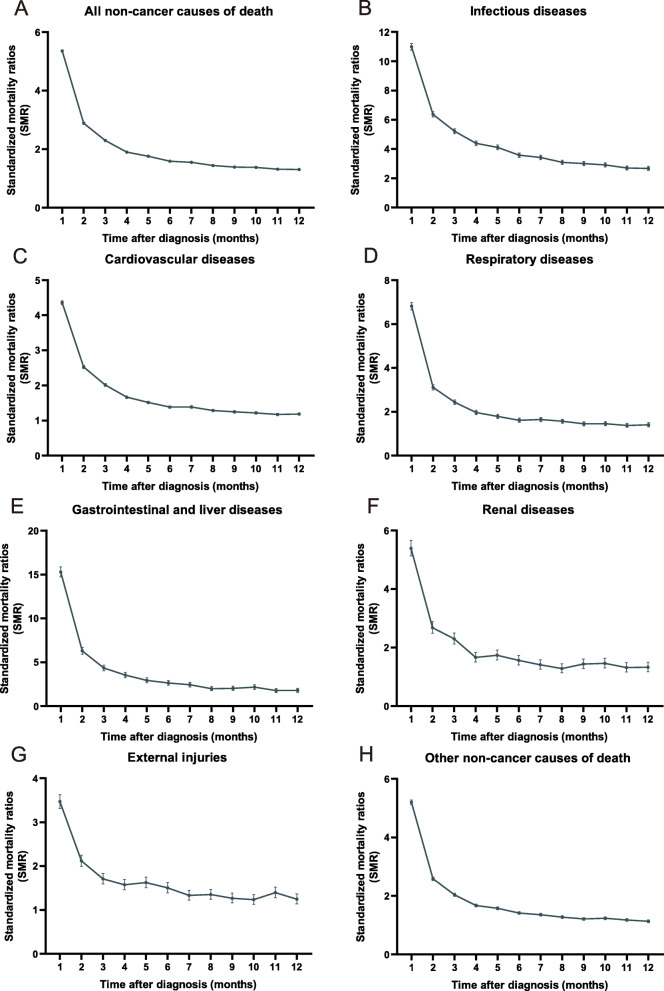
Risk of deaths from major non-cancer causes by time from diagnosis

**Fig. 3:**
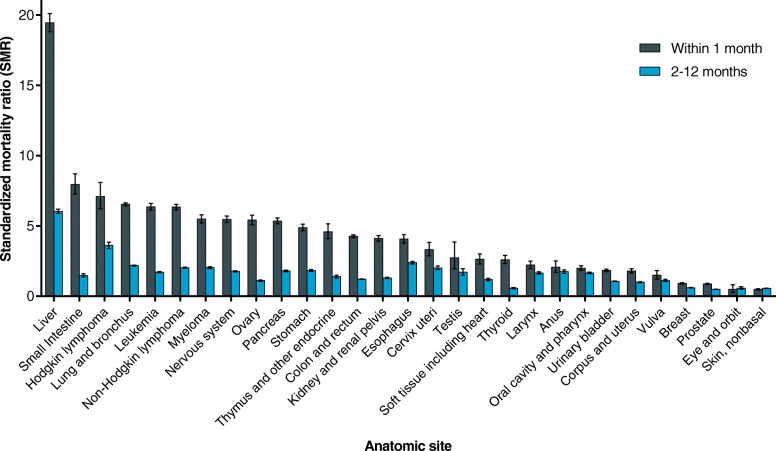
Risk of non-cancer deaths within the first year after diagnosis by anatomic site and time from diagnosis

The trends of SMR for non-cancer deaths during follow-up after diagnosis were determined in this cohort (Fig. 2 and Fig. 3). For all causes and sites combined, the highest risk elevation was observed within the first month of diagnosis, and a rapid decrease in the risks was observed during the first-year follow-up. Similar trends were observed in all major non-cancer causes (Fig. 2). In site-specific analysis, similar trends were found in most sites, with only a few exceptions (cancers of the anus, non-basal skin, and testis) (Fig. 3).

### Anatomic site-specific analysis for the major types of non-cancer causes of death

**Fig. 4:**
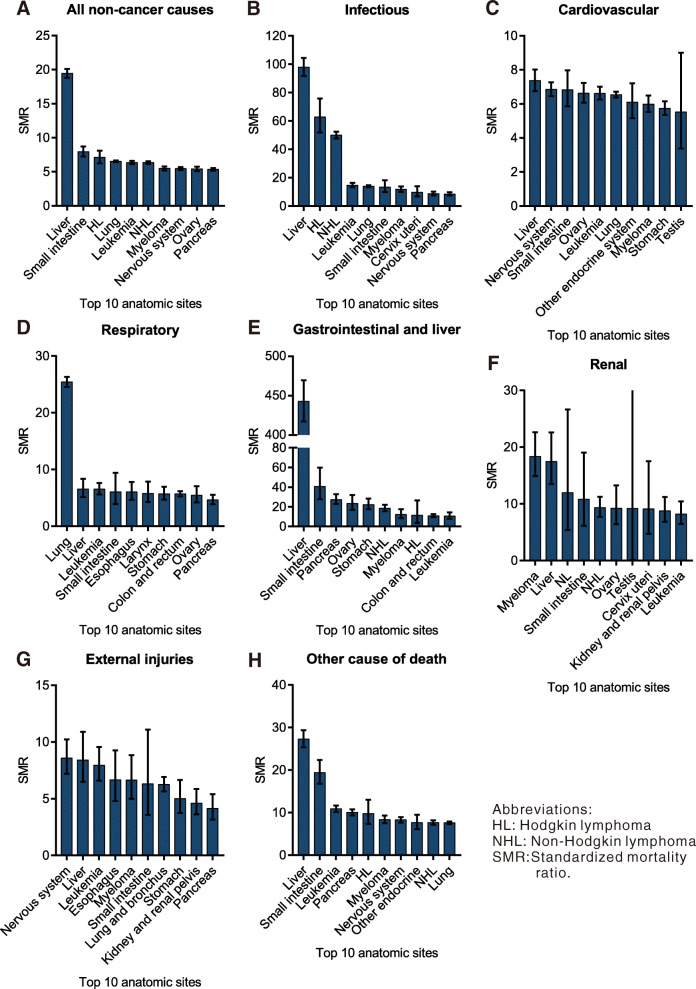
Risk of deaths from major non-cancer causes within the first year after diagnosis by anatomic site

For the major types of non-cancer deaths, the site-specific analyses are presented in Fig. 4. In terms of mortality from infectious diseases, the SMRs were the highest among those with liver cancer (SMR: 47.03; 95% CI: 45.29–48.84), lymphomas (SMR: 24.68; 95% CI: 24.09–25.27), and anal cancer (SMR: 12.27; 95% CI: 10.89–13.82). The highest SMRs for CVD after a cancer diagnosis were observed for brain cancer (SMR: 5.00; 95% CI: 4.73–5.28), followed by liver (SMR: 4.24; 95% CI: 4.04–4.44) and lung cancer (SMR: 3.79; 95% CI: 3.74–3.85). Among all types of cancer, lung cancer was associated with the highest SMRs for respiratory diseases (SMR: 10.92; 95% CI: 10.70–11.15), followed by laryngeal cancer (SMR: 4.83; 95% CI: 4.32–5.39) and esophageal cancer (SMR: 4.63; 95% CI: 4.13–5.19). The SMRs for gastrointestinal and liver diseases were remarkably high for those with liver cancer (SMR: 162.60; 95% CI: 156.70–168.72), pancreatic cancer (SMR: 9.33; 95% CI: 8.16–10.68), and stomach cancer (SMR: 7.21; 95% CI: 6.15–8.44). For renal diseases, the SMRs were the highest among patients with myelomas (SMR: 9.25; 95% CI: 8.33–10.27), liver cancers (SMR: 7.66; 95% CI: 6.56–8.93), cervical cancer (SMR: 4.99; 95% CI: 3.67–6.77), and renal cancer (SMR: 4.69; 95% CI: 4.17–5.26). The SMRs for external injuries were remarkably higher among subjects with cancers of the liver (SMR: 4.75; 95% CI: 4.17–5.40), esophagus (SMR: 4.44; 95% CI: 3.82–5.16) and lung (SMR: 3.77; 95% CI: 3.59–3.97).

## Discussion

In our population-based cohort study including more than 7 million patients with cancer, the risk of non-cancer deaths was particularly increased within the first year after diagnosis; mortality was approximately 2.34-fold that of the general population. The majority of non-cancer deaths were caused by CVD, infectious diseases, and respiratory diseases. When compared with the normal population, the risk elevations from infectious diseases were highest in all causes. Patients with liver, brain, and lung cancer are most likely to die of non-cancer causes in the first year. The risk of non-cancer deaths was highest in the period immediately following a cancer diagnosis and decreased rapidly.

The majority of non-cancer causes of death within the first year may be largely classified into two groups: chronic comorbidities and acute infections. The most common cause of death from chronic comorbidities is CVD, which mainly includes heart and cerebrovascular diseases (Table 2). We also found that patients with cancer often had accompanying chronic comorbid conditions in the same or adjacent sites. We noted an increase in the risk of death from respiratory diseases (mainly chronic obstructive pulmonary disease [COPD]) in patients with lung cancers. In addition, the risk of deaths from gastrointestinal and liver diseases was highest in the diseases of the digestive organs, including the liver, pancreas, stomach and esophagus (Fig. 4). This may be attributed to the shared causal factors between the comorbid conditions and cancers, particularly in the same or adjacent sites. Lung cancer and COPD, for example, are all caused or aggravated by smoking [15]. In addition, chronic inflammation has been postulated as the obvious culprit linking COPD and lung cancer [16].

As the second predominant cause of death among patients with cancer after a cancer diagnosis, fatal infections have been interpreted to be a consequence of the immunosuppression induced by the effects of the malignancy itself and by various modern cancer therapies, including chemotherapeutic regimens, hematopoietic stem cell transplantation, invasive procedures, or medical devices and malnutrition [17–20]. Consistent with our previous findings, cancer patients had greater risks of dying from infections, and the degree of the risks was positively correlated with the stages of cancer [21, 22]. In our analyses, the particularly high risk of death from infectious diseases in patients with liver cancer may be attributable to its association with acute or chronic viral hepatitis; this elucidated the important role of viral hepatitis in both the development of and mortality from liver cancer [23, 24]. As shown in Fig. 4, elevated SMRs for infectious diseases were also observed in nearly all hematological malignancies, including lymphomas, leukemias, and myelomas; this reflected the substantial risks of immune-compromise induced by hematologic malignancies and iatrogenic immunosuppression caused by systemic chemotherapy and hematopoietic stem cell transplantation [25, 26]. Furthermore, the notably high SMRs in patients with lymphomas probably reflects its relation with human immunodeficiency virus (HIV) infection, particularly since our study covered the period of increase in the burden of HIV infection [27, 28]. The findings of our study indicate the underlying interactive correlation between infection and cancer. Immunosuppression induced by cancer and its treatments may play an important role in infectious disease complications. Conversely, previous studies also revealed the important role that infectious agents play in carcinogenesis [17, 18, 29, 30]. Therefore, strategies to control infectious diseases, both before and after a cancer diagnosis, should be optimized. Following accumulation of all non-cancer causes, the risk of non-cancer deaths was highest in patients with liver cancers. As the third leading cause of cancer-related mortality worldwide, the poor prognosis of liver cancer may have contributed considerably to these results [31]. Additionally, liver cancer and the non-cancer co-morbidities share certain potential risk factors (modifiable and non-modifiable). As described previously, in addition to the strong association between acute or chronic viral hepatitis with the development of liver cancer, they were also related with the particularly high risks of infectious disease complications in these patients [23, 24]. Consequently, the mortalities of both chronic liver disease and infectious disease complications were substantially elevated in patents with liver cancer. The high mortality from heart disease in patients with liver cancer may be attributed to the shared causal factors among these conditions, including increasing age, male sex, diet, alcohol abuse, tobacco smoking, and fatty and nonalcoholic fatty liver disease [32–35].

Among the different types of cancer, lung cancer was responsible for the highest number of non-cancer deaths, and also had a significantly elevated SMR. Lung cancer is the most common malignancy and the leading cause of death worldwide [31]. In this study, we found that lung cancer was also a leading cause of death involving non-cancer comorbidities in the first year following diagnosis. Besides, the risk factors of lung cancer, such as age and smoking, are also strongly associated with non-cancer comorbidities, including diseases of the cardiovascular, pulmonary and other systems [36].

Interestingly, the risk of death from renal diseases was markedly high in patients with myeloma. Renal insufficiency in myeloma patients is mainly caused by renal impairment resulting from the accumulation and precipitation of homogeneous immunoglobulins or light chains; the predominant renal pathology is cast nephropathy [37]. Renal involvement may manifest as acute or chronic renal failure, nephrotic syndrome, non-nephrotic proteinuria, or tubular function defects [37, 38].

The main strength of our study was that it included a population-based cohort and comprehensively obtained the diagnostic confirmation of cancers and their fatal outcomes. However, there were several limitations. First, the causes of death may have been potentially misclassified due to inaccurate coding in the death certificates [39]. However, previous studies have shown that for several non-cancer causes of death, including coronary heart disease [40] and suicide [41, 42], the cause of death codes obtained from death certificates are reasonably valid (sensitivity and specificity > 70%) compared to the gold standard assessment of the cause of death [40, 43]. In addition, systematic and standardized data collection procedures are used to ensure that the causes of death recoded in the SEER are accurate [44].

Second, in view of maintaining confidentiality, deaths from HIV and other infections, including viral hepatitis and parasitic diseases are incorporated under a single code in the SEER database [7]. Therefore, the direct contribution of deaths from HIV, viral hepatitis, and other infections, to the increase of SMR in patients with cancer could not be accurately assessed.

Third, the SEER database does not provide detailed information on cancer treatment; this precluded the analysis of non-cancer mortality based on receipt of specific chemotherapy drugs and/or doses of chemotherapy or radiotherapy.

Finally, personal information on family history or genetics was not available in the SEER database, thus our analysis could not provide insight on the role of heredity in risk of non-cancer deaths.

## Conclusions

Risks from non-cancer deaths significantly increase in the first year following a cancer diagnosis in comparison with the general population, particularly in the first month. Risks of non-cancer deaths vary by the types of causes and anatomic sites of cancer. Our data underscore the importance of comprehensive and early multidisciplinary care for mitigating such risks.

## Data Availability

The datasets generated and analyzed during the current study are available in the SEER repository (https://seer.cancer.gov/seerstat/).

## Abbreviations

SEER: Surveillance, Epidemiology, and End Results
SMR: Standardized Mortality Ratios
CI: Cofidence Interval
CVD: Cardiovascular Disease
